# A Multimethodological Characterization of *Cannabis sativa* L. Inflorescences from Seven Dioecious Cultivars Grown in Italy: The Effect of Different Harvesting Stages

**DOI:** 10.3390/molecules26102912

**Published:** 2021-05-14

**Authors:** Mattia Spano, Giacomo Di Matteo, Cinzia Ingallina, Bruno Botta, Deborah Quaglio, Francesca Ghirga, Silvia Balducci, Silvia Cammarone, Enio Campiglia, Anna Maria Giusti, Giuliana Vinci, Mattia Rapa, Salvatore Ciano, Luisa Mannina, Anatoly P. Sobolev

**Affiliations:** 1 Department of Chemistry and Technology of Drugs, Sapienza University of Rome, Piazzale Aldo Moro 5, 00185 Rome, Italy; mattia.spano@uniroma1.it (M.S.); giacomo.dimatteo@uniroma1.it (G.D.M.); cinzia.ingallina@uniroma1.it (C.I.); bruno.botta@uniroma1.it (B.B.); deborah.quaglio@uniroma1.it (D.Q.); francesca.ghirga@uniroma1.it (F.G.); silvia.balducci@uniroma1.it (S.B.); silvia.cammarone@uniroma1.it (S.C.); 2 Department of Agricultural and Forest Sciences, University of Tuscia, Via San Camillo de Lellis snc, 01100 Viterbo, Italy; campigli@unitus.it; 3 Department of Experimental Medicine, Sapienza University of Rome, P.le Aldo Moro 5, 00185 Rome, Italy; annamaria.giusti@uniroma1.it; 4 Department of Management, Sapienza University of Rome, Via del Castro Laurenziano 9, 00161 Rome, Italy; giuliana.vinci@uniroma1.it (G.V.); mattia.rapa@uniroma1.it (M.R.); salvatore.ciano@uniroma1.it (S.C.); 5 Institute for Biological Systems, Magnetic Resonance Laboratory “Segre-Capitani”, CNR, Via Salaria Km 29.300, 00015 Monterotondo, Italy; anatoly.sobolev@cnr.it

**Keywords:** industrial hemp, dioecious cultivars, inflorescences, phenological growth stages, cannabinoids, metabolite profile, antioxidant activity, multimethodological analysis

## Abstract

The chemical profile of the female inflorescence extracts from seven *Cannabis sativa* L. dioecious cultivars (Carmagnola, Fibranova, Eletta Campana, Antal, Tiborszallasi, Kompolti, and Tisza) was monitored at three harvesting stages (4, 14, and 30 September), reaching from the beginning of flowering to end of flowering/beginning of seed formation, using untargeted nuclear magnetic resonance (NMR) and targeted (ultra-high-performance liquid chromatography (UHPLC) and spectrophotometry) analyses. The tetrahydrocannabinol content was always below the legal limits (<0.6%) in all the analyzed samples. The NMR metabolite profile (sugars, organic acids, amino acids, and minor compounds) subjected to principal components analysis (PCA) showed a strong variability according to the harvesting stages: samples harvested in stage I were characterized by a high content of sucrose and myo-inositol, whereas the ones harvested in stage II showed high levels of succinic acid, alanine, valine, isoleucine, phenylalanine, and threonine. Samples harvested in stage III were characterized by high levels of glucose, fructose, choline, trigonelline, malic acid, formic acid, and some amino acids. The ratio between chlorophylls and carotenoids content indicated that all plants grew up exposed to the sun, the Eletta Campana cultivar having the highest pigment amount. Tiborszallasi cultivar showed the highest polyphenol content. The highest antioxidant activity was generally observed in stage II. All these results suggested that the *Cannabis sativa* L. inflorescences of each analyzed dioecious hemp cultivar presented a peculiar chemical profile affected by the harvesting stage. This information could be useful for producers and industries to harvest inflorescences in the appropriate stage to obtain samples with a peculiar chemical profile suitable for proper applications.

## 1. Introduction

*Cannabis sativa* L. is a plant of the *Cannabaceae* family widely used all over the world thanks to its multipurpose applications and climatic and territorial adaptability. From a taxonomic point of view, different classifications of *Cannabis sativa* L. varieties exist causing sometimes some confusion. However, the generally accepted botanical classification is based on the content of psychotropic (Δ9-tetrahydrocannabinol, THC) and non-psychotropic (cannabidiol, CBD) cannabinoids: drug-type chemotype is characterized by higher levels of THC with respect to CBD, whereas fiber-type chemotype is characterized by low levels of THC (usually under 0.2%) and high levels of CBD [1]. Industrial hemp is a fiber-type *Cannabis sativa* L. traditionally cultivated and used worldwide for fiber (production of paper, ropes, and clothes) and seed (food uses) production [2]. During the 70′s, the illicit use of drug-type *Cannabis* for narcotic purposes led the European authorities to strongly restrict the cultivation and the industrial applications of *Cannabis sativa* L., including industrial hemp. In Italy, the second greatest hemp producer in the world after Russia [3], hemp cultivation almost disappeared due to legislative restrictions and the economic crisis. In the 90′s, the European Union published new regulations to promote and reintroduce the cultivation of *Cannabis sativa* L. cultivar with a low THC content (≤0.2%, with a tolerance value of 0.6%) [4]. These cultivars are listed in an official European database [5], and their THC content is constantly monitored.

Following the European guidelines, Italy has started a process for the valorization of hemp cultivation and applications, and in 2017, the Lazio Region (Central Italy) approved a new regulation to enhance the reintroduction and the growth of *C. sativa* cultivations in the regional areas. In this scenario, the necessity of a better characterization and development of local *Cannabis sativa* L. products prompted the Lazio Region to support a project titled “Industrial Hemp: development and enhancement of a new eco-sustainable agro-food chain”. *Cannabis* inflorescences have been traditionally considered as a waste product in the industrial field but, in the last years, the interest for this part of the plant increased exponentially, mainly for the extraction of cannabinoids and essential oils [6,7,8].

The chemical composition of hemp inflorescences can be affected by several factors such as genotype, pedoclimatic conditions, agronomical practices, and harvesting time [9,10,11,12].

*Cannabis* is naturally a dioecious cultivar; namely, the male and female flowers are presented on different plants: pollination occurs with the wind. Monoecious *Cannabis* cultivars are also obtained through breeding selection. The dioecious ones are mainly used for fiber production, whereas the monoecious ones for seed production [13,14].

In a previous paper [12], the chemical composition of the inflorescences from four *Cannabis sativa* L. monoecious cultivars, namely Ferimon, Uso-31, Felina 32, and Fedora 17, recently introduced in the Lazio Region, was monitored over the season using a multimethodological approach [15,16].

In this paper, the proposed protocol was extended to dioecious female inflorescences of seven *Cannabis sativa* L. cultivars (Carmagnola, Fibranova, Eletta Campana, Antal, Tiborszallasi, Kompolti, and Tisza) to determine their chemical composition and monitor possible variations according to three harvesting stages. Targeted methodologies, namely ultra-high-performance liquid chromatography (UHPLC) and spectrophotometry, were applied to analyze and to monitor cannabinoids, natural pigments, and total phenolics, as well as to evaluate the antioxidant activity of the inflorescences extracts. The seasonal time trend of sugars, amino acids, organic acids, and minor compounds was monitored using untargeted NMR spectroscopy, a very powerful tool to investigate the metabolite profile of vegetable matrices [17,18].

## 2. Results

Targeted and untargeted methodologies were used to determine the chemical profile of the inflorescences extracts at three harvesting stages (4 September, 14 September, and 30 September).

### 2.1. Targeted Analyses: Cannabinoids, Chlorophylls, Total Carotenoids, and Phenolics Content, Antioxidant Activity

The cannabinoid profile of the inflorescences methanol extracts was obtained using UHPLC. The concentrations of eight cannabinoids, namely CBDV, CBD, CBC, CBDA, CBG, CBN, Δ9-THC, and Δ9-THCA, are reported in Table 1. Δ8-THC, previously reported in medical cannabis samples [19], was not detected in the investigated samples. The total Δ9-THC/total CBD ratio was lower than 1.0 for all the analyzed samples, according to the fiber-type chemotype of all the selected hemp cultivars in the present study [20,21].

The chloroform extracts of *Cannabis sativa* L. inflorescences were analyzed with spectrophotometric technique in order to quantify the amount of chlorophyll a, chlorophyll b, and total carotenoids (Table 2). Chl a/chl b and chl (a+b)/carotenoid ratios are also reported in Table 2. Chl a/chl b ratio could be used as an indicator of the light exposition of the plants: a value of 1.1–1.3 was characteristic of shade exposition, whereas higher values were typical for sun-exposed plants. Chl (a+b)/car ratio was used as an indicator of both sun exposition and stress or senescence conditions of plants: a value of 4.2–5 was generally typical of sun-exposed plants, whereas a value below 3.5 was an indicator of senescence progress [22].

Total phenolics content and antioxidant activity (DPPH and ABTS assays) of hemp inflorescences (Table 3) were determined by spectrophotometric analysis. These untargeted assays were easy to perform, rapid, low-cost, and useful for achieving an overview of the antioxidant activity of the samples [23]. The ABTS•+ molecule showed low selectivity with aromatic OH- groups that gave a significant antioxidant contribution. In contrast, DPPH• (which was more selective with these compounds) had the disadvantage of steric accessibility. Small molecules had better access to the radical site showing higher apparent antioxidant activity [24]. For these reasons, the integrated use of these assays could complete the TPC assay’s information. Hemp inflorescences exhibited high variability in antioxidant activity and total phenolic content.

### 2.2. Untargeted Analysis of Metabolite Profile by NMR and PCA

**Figure 1:**
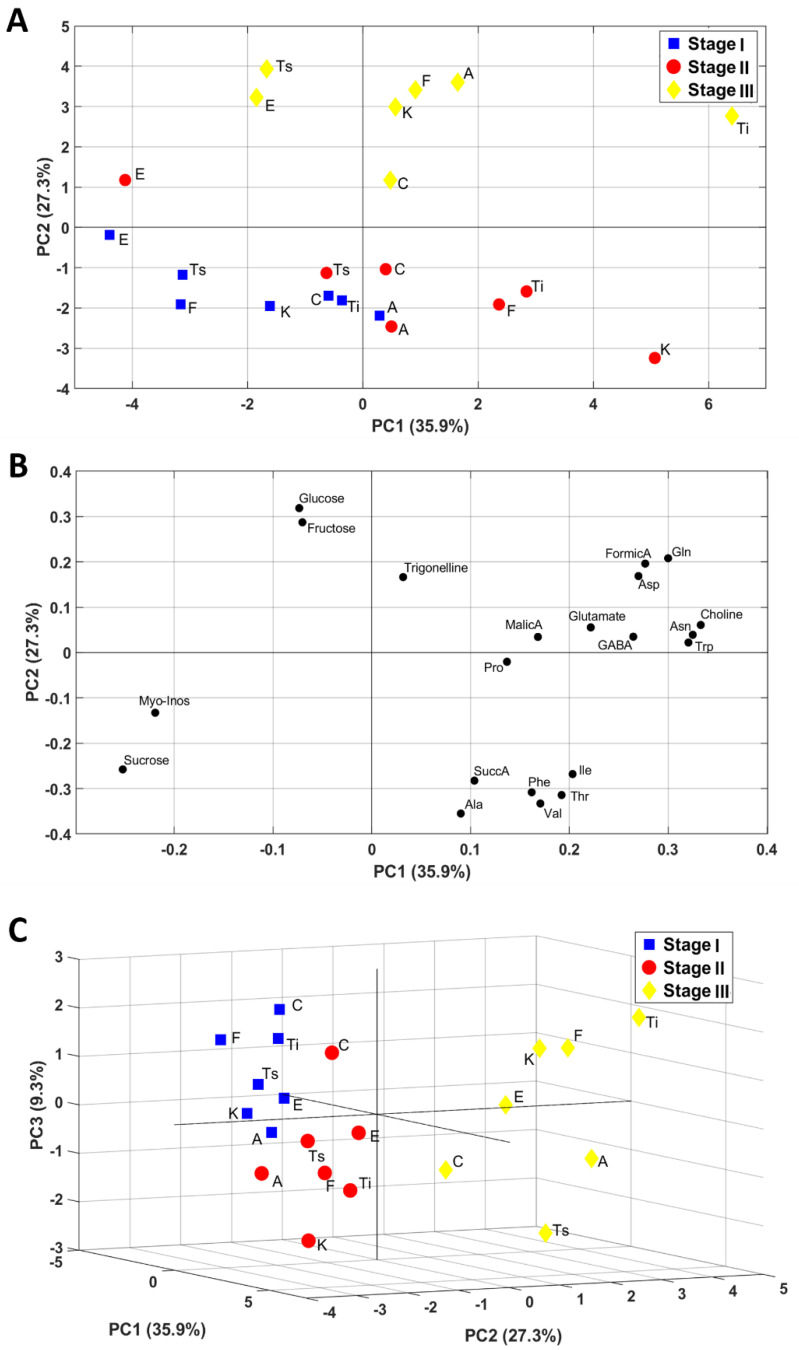
Principal components analysis (PCA) maps obtained using NMR data of inflorescences samples: (**A**) scores plot and (**B**) loadings plot relative to PC1 and PC2 (PCs); (**C**) score plot relative to PC1, PC2, and PC3. PC1, PC2, and PC3 represent 35.9%, 27.3%, and 9.3% of the total variance, respectively.

The NMR profile of hydroalcoholic Bligh–Dyer inflorescences extracts was obtained by using literature data [12]. Six sugars, six organic acids, thirteen amino acids, choline, and trigonelline were quantified at the three harvesting stages, and used for multivariate PCA, see Figure 1. In Table 4, the quantified metabolites in hydroalcoholic Bligh–Dyer extracts are listed, together with the chemical shift (ppm) of the signals used for quantification. Figure 1A,B show 2D PCA plots of sample scores and loading (PC1 versus PC2), whereas Figure 1C shows a 3D PCA plot of PC1, PC2, and PC3 sample scores. The first two PCs accounted for 63.2% of the variability within the data, whereas the first three PCs provided for 72.5% of the variability. PCs score plots showed a sample groping according to the harvesting stages. In particular, as it was clearly shown in the 3D score plot, the first two harvesting stages were separated along PC1 and PC3 (stage I having mainly negative PC1 values and positive PC3 values, stage II having positive PC1 values and negative PC3 values). Stage III (30 September) was well separated from the others and characterized by positive values of PC2. The contribution of the variables to this grouping given by the variable loadings reported in Figure 1B was discussed in detail in the Discussion paragraph.

### 2.3. Metabolite Profile of the Inflorescences at Three Harvesting Stage

Trends of metabolite profiles during the three harvesting stages were discussed separately for each cultivar. Finally, a comparison among cultivars was also reported.

#### 2.3.1. Carmagnola

Cannabinoids. CBD, the main cannabinoid, showed a gradual increase over the three different harvesting stages, reaching its maximum in stage III. The psychotropic cannabinoid THC remained constant over time, with a small decrease in stage III. The highest value of CBG was measured in stage II, whereas for CBC, the highest value was detected in stage I. CBDA was detected only in the first two stages, whereas THCA, CBDV, and CBN were not detected in all the harvesting stages (Table 1).

**Figure 2:**
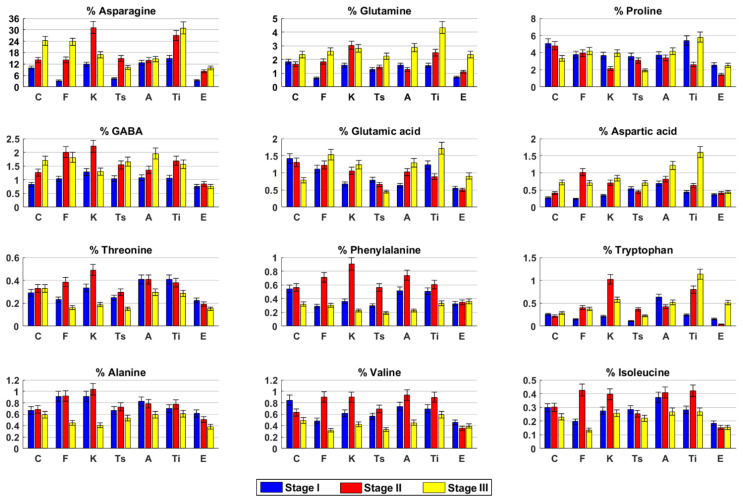
Free amino acids content in hydroalcoholic extracts of *Cannabis sativa* L. fresh inflorescences at three harvesting stages. C (Carmagnola), F (Fibranova), K (Kompolti), Ts (Tisza), A (Antal), Ti (Tiborszallasi), E (Eletta Campana).

Free amino acids. Asparagine was found to be the main amino acid in hemp inflorescences. Asparagine amount, together with GABA and aspartic acid, increased during the time (Figure 2). Valine, glutamic acid, and proline showed an opposite trend. Glutamine content was quite constant in the first two harvesting stages and then slightly increased, whereas phenylalanine and isoleucine slightly decreased in stage III. Alanine and tryptophan concentrations were always constant.

**Figure 3:**
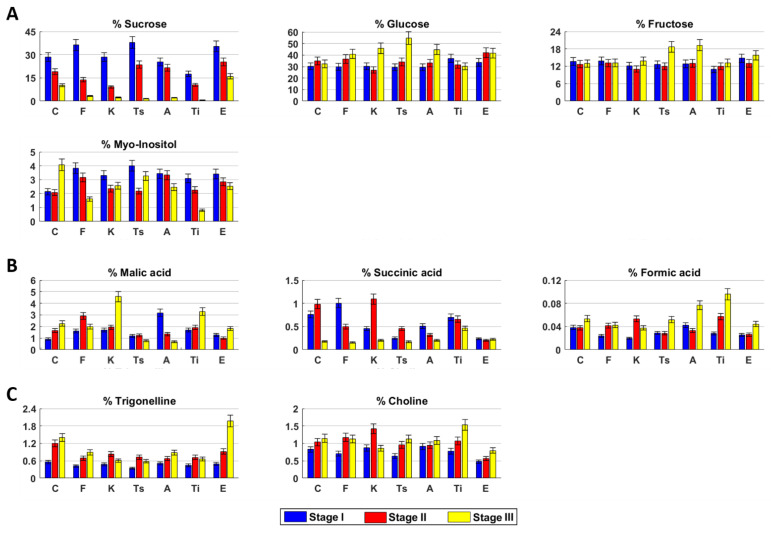
Sugars (**A**), organic acids (**B**), and miscellaneous compounds (**C**) content in hydroalcoholic extracts of *Cannabis sativa* L. fresh inflorescences at different harvesting stages. C (Carmagnola), F (Fibranova), K (Kompolti), Ts (Tisza), A (Antal), Ti (Tiborszallasi), E (Eletta Campana).

Sugars. Sucrose, glucose, and fructose were the main sugars in hemp inflorescences. Sucrose drastically decreased from stage I to stage III (Figure 3A). Glucose and fructose remained quite constant. Myo-inositol content was constant in the first two stages and then doubled.

Organic acids. Malic acid increased over time, whereas succinic acid increased until stage II and then drastically decreased (Figure 3B). Formic acid was constant in the first two stages and then slightly increased.

Miscellaneous compounds. Trigonelline and choline increased over time (Figure 3C).

Pigments. Chlorophyll a, chlorophyll b, and total carotenoid contents decreased over time (Table 2). Chl a/chl b and chl (a+b)/car ratio values at the three stages were typical of sun-exposed plants, with the highest values in stage II.

Total phenolics and antioxidant activity. The highest content of total phenolic compounds (TPC) was measured in stage II, whereas the lowest one was found in stage III (Table 3). The same trend was also observed for both the inhibition rates in DPPH and ABTS assays.

#### 2.3.2. Fibranova

Cannabinoids. The highest contents of CBD and THC were measured in stage II (Table 1). Among the other cannabinoids, CBDA, CBG, and CBC were present only in the first two stages. Moreover, CBDV and CBN were detected only in stage II and stage III, respectively. THCA was never detected in all stages considered.

Free amino acids. Asparagine, glutamine, proline, and glutamic acid contents increased over time (Figure 2). Tryptophan and GABA increased from stage I to stage II and remained constant, whereas alanine was constant until stage II and then decreased. All the other amino acids analyzed reached their highest content in stage II.

Sugars. Sucrose and myo-inositol decreased during the time, whereas glucose showed an opposite trend. Fructose content was constant over time (Figure 3A).

Organic acids. The highest content of malic acid was measured in stage II, whereas succinic acid decreased over time. Formic acid slightly increased in stage II and then remained constant (Figure 3B).

Miscellaneous compounds. Choline and trigonelline concentrations increased from stage I to stage II and then remained constant (Figure 3C).

Pigments. Chlorophyll a, chlorophyll b, and total carotenoids content reached their highest value in stage II. Chl a/chl b and chl (a+b)/car ratio values at the three stages were typical of sun-exposed plants, with the highest value of chl a/chl b in stage I and the highest value of chl (a+b)/car in stage II (Table 2).

Total phenolics and antioxidant activity. The highest content of TPC was measured in stage II, whereas the lowest one was found in stage I. The same trend was also observed for the inhibition rate (I%) of ABTS assay, whereas in the DPPH assay, the highest and the lowest I% values were measured in stage I and stage III, respectively (Table 3).

#### 2.3.3. Kompolti

*Cannabinoids*. The highest contents of THC, CBD, and CBC were measured in stage II, whereas for CBG, the highest value was measured in stage III. As a common trend, CBDA was not measured in stage III. THCA, CBDV, and CBN were never detected (Table 1).

*Free amino acids*. Asparagine, GABA, valine, isoleucine, threonine, phenylalanine, and tryptophan reached their highest concentrations in stage II. Conversely, in this stage, the lowest value of proline was measured. Glutamine, glutamic acid, and aspartic acid increased in stage II and remained constant (Figure 2).

*Sugars*. Sucrose drastically decreased over time (Figure 3A). Glucose and fructose slightly decreased in stage II and then increased. Myo-inositol decreased in stage II and then remained quite constant.

*Organic acids*. Malic acid slightly increased from stage I to stage II and then drastically decreased. Succinic and formic acids reached their highest content in stage II. (Figure 3B).

*Miscellaneous compounds*. Both choline and trigonelline reached their highest amount in stage II. (Figure 3C).

*Pigments*. Chlorophyll a, chlorophyll b, and total carotenoids, together with chl a/chl b and chl (a+b)/car ratios, decreased over time (Table 2).

*Total phenolics and antioxidant activity*. The highest content of TPC was measured in stage II, whereas the lowest one was found in stage III (Table 3). The same trend was also observed for both I % in DPPH and ABTS assays, although I % in the DPPH assay was quite the same in the first two harvesting stages.

#### 2.3.4. Tisza

*Cannabinoids*. CBD drastically increased from stage I to stage III. The highest THC content was measured in stage II and drastically decreased (four times less) in stage III, whereas CBG content decreased during the time (Table 1). CBC and CBDA were measured only in stage II, whereas THCA, CBDV, and CBN were not detected.

*Free amino acids*. Asparagine, valine, threonine, phenylalanine, and tryptophan reached their highest contents in stage II, whereas proline, glutamic acid, and isoleucine slightly decreased over time (Figure 2). Glutamine and aspartic acid amounts were quite constant until stage II and then slightly increased. GABA increased in stage II and then remained constant, whereas the alanine amount was constant until stage II and then slightly decreased.

*Sugars*. Sucrose content drastically decreased over time, whereas glucose had an opposite trend (Figure 3A). Fructose content was constant until stage II and then increased, whereas myo-inositol content decreased in stage II and then increased.

*Organic acids*. Malic acid and formic acid contents were constant until stage II, then decreased and increased, respectively. Succinic acid amount was the highest in stage II (Figure 3B).

*Miscellaneous compounds*. Trigonelline content increased in stage II and then remained constant, whereas choline increased over time. (Figure 3C).

*Pigments*. Chlorophyll a, chlorophyll b, and total carotenoids, together with chl (a+b)/car, reached their highest value in stage II, whereas chl a/chl b ratio value was the highest in stage I (Table 2). However, both ratios were typical of sun-exposed plants.

*Total phenolics and antioxidant activity*. The highest content of TPC was measured in stage II, whereas the lowest one was found in stage III (Table 3). The same trend was also observed for both I% in DPPH and ABTS assays.

#### 2.3.5. Antal

*Cannabinoids*. THC, CBD, and CBG reached their highest values in stage I and then decreased. Similarly, also the highest CBDA content was measured in stage I, although it was not detected in stage III. CBDV and CBC were detected only in stage III, whereas THCA and CBN were not detected in the stages considered (Table 1).

*Free amino acids*. Asparagine, GABA, glutamic acid, and aspartic acid slightly increased over time, whereas alanine had an opposite trend. Glutamine, proline, and tryptophan slightly decreased in stage II and then increased, whereas valine, isoleucine, and phenylalanine reached their highest content in stage II (Figure 2). Threonine remained constant until stage II and then decreased.

*Sugars*. Sucrose content drastically decreased over time, whereas glucose slightly increased (Figure 3A). Fructose and myo-inositol remained constant until stage II and then increased and decreased, respectively.

*Organic acids*. Malic and succinic acids decreased over time, whereas formic acid slightly decreased in stage II and then increased (Figure 3B).

*Miscellaneous compounds*. Trigonelline content was quite constant over time, whereas choline slightly increased. (Figure 3C).

*Pigments*. Chlorophyll a and total carotenoids content decreased over time, whereas chlorophyll b remained constant in the first two stages and then decreased. The highest values of chl a/chl b and chl (a+b)/car ratios were measured in stage II (Table 2).

*Total phenolics and antioxidant activity*. The highest values of TPC and I% in both DPPH and ABTS assays were measured in stage I then decreased over time (Table 3).

#### 2.3.6. Tiborszallasi

*Cannabinoids*. THC, CBD, CBDA, and CBC reached their highest content in stage II, whereas the CBG amount decreased over time. THCA was detected only in stage II, whereas CBDV and CBN were never detected (Table 1).

*Free amino acids*. Asparagine, glutamine, aspartic acid, and tryptophan increased over time, whereas threonine had an opposite trend (Figure 2). Proline and glutamic acid reached their lowest content in stage II. Conversely, in this stage, the highest concentrations of GABA, alanine, valine, isoleucine, and phenylalanine were found.

*Sugars*. Sucrose, glucose, and myo-inositol concentrations decreased over time, whereas fructose had an opposite trend (Figure 3A).

*Organic acids*. Malic and formic acids increased over time, whereas succinic acid had an opposite trend (Figure 3B).

*Miscellaneous compounds*. Trigonelline increased in stage II and then remained constant. Choline slightly increased over time (Figure 3C).

*Pigments*. Chlorophyll a, chlorophyll b, and total carotenoids, together with chl (a+b)/car ratio, increased in stage II and then decreased (Table 3). Chl a/chl b ratio reached its highest content in stage I. Chl (a+b)/car ratio value in stage III indicated that the hemp plants were in the early stage of the senescence process.

*Total phenolics and antioxidant activity*. The highest content of TPC was measured in stage II, whereas the lowest one was found in stage I. The same trend was also observed for I% of the DPPH assay, whereas in the ABTS assay, the highest and the lowest I% values were measured in stage II and stage III, respectively (Table 4).

#### 2.3.7. Eletta Campana

*Cannabinoids*. The highest contents of THC, CBD, CBDA, and CBC were measured in stage II, although CBDA and CBC were not detected in stage III and stage I, respectively (Table 1). CBN was measured only in stage III, whereas THCA and CBDV were never detected.

*Free amino acids*. Asparagine and glutamine increased over the stages, whereas alanine and threonine showed an opposite trend (Figure 2). Proline, glutamic acid, valine, isoleucine, and tryptophan contents decreased in stage II and then increased. GABA, aspartic acid, and phenylalanine amounts remained quite constant over time.

*Sugars*. Sucrose and myo-inositol decreased over time, whereas glucose increased until stage II and then remained constant (Figure 3A). Fructose content had its highest concentration in stage II.

*Organic acids*. Malic acid content slightly decreased in stage II and then increased, whereas succinic acid remained quite constant during the three stages (Figure 3B). Formic acid concentration remained constant until stage II and then increased.

*Miscellaneous compounds*. Trigonelline and choline increased over time. (Figure 3C).

*Pigments*. Chlorophyll a, chlorophyll b, and total carotenoids decreased over time (Table 3). The highest values of chl a/chl b and chl (a+b)/car ratios were found in stage I and stage II, respectively. However, the ratio values in all the stages were indicators of the light exposition of the plant.

*Total phenolics and antioxidant activity*. The highest TPC content was measured in stage II, whereas the lowest one was found in stage I. The same trend was also observed for I% of ABTS assay, whereas in the DPPH assay, the highest and the lowest I% values were measured in stage II and stage III, respectively (Table 4).

## 3. Discussion

The cannabinoids content of the seven investigated cultivars (Carmagnola, Fibranova, Kompolti, Tisza, Antal, Tiborszallasi, and Eletta Campana) revealed common features but also important differences. In all the analyzed cultivars, the THC content was always under the legal limit, including Carmagnola, Fibranova, and Antal cultivars that, in 2019, were excluded from the European Plant Database because of the variable THC content. In particular, the lowest THC concentrations were measured in stage III in Tisza and Eletta Campana cultivars, whereas the highest one was measured in Tiborszallasi in stage II (0.51%). As expected for the fiber-type chemotype, CBD was found to be the most abundant cannabinoid, with the highest concentration measured in Kompolti cultivar in stage II (5.09%). The presence of high levels of CBD in female inflorescences of dioecious *Cannabis sativa* L. can be important from a pharmacological/nutraceutical point of view, considering that CBD is a non-psychotropic cannabinoid with several biological activities [25]. CBDA, the acid form of CBD, was present only in stage I and stage II, whereas THCA, the acid form of THC, was measured only in Tiborszallasi cultivar in stage II. Similarly, CBN was measured only in stage III in Fibranova and Eletta Campana cultivars, whereas CBDV was measured only in Fibranova and Antal cultivars in stage II and stage III, respectively. CBG and CBC were detected in most of the samples, as previously reported. Although these two cannabinoids were generally of less interest with respect to THC and CBD, they represented a new potential resource for the pharmaceutical/nutraceutical field [26,27].

In a previous HPLC study [21], the cannabinoids content of ethanolic extracts from Tisza, Tiborszallasi, Antal, and Carmagnola cultivars grown in Slovenia in September was reported: lower levels of THC and CBD with respect to those of THCA and CBDA had been detected. On the contrary, in our study, the neutral forms CBD and THC were more abundant than their acid forms: THCA was observed only in one sample, whereas CBDA was present only in stage I and stage II of all cultivars, confirming a decrement in this cannabinoid over the harvesting time. Therefore, the different contents of THCA and CBDA detected in the inflorescences grown in Slovenia were potentially due to different agronomic growing conditions.

In another study, the THC and CBD contents in hexane extracts of Kompolti air-dried inflorescences grown in Austria [28] were reported being at least two times lower than those observed in the same cultivar in the present study. However, no indication regarding the harvesting stage was reported, making it difficult for comparison.

The spectrophotometric analysis of chlorophylls and total carotenoids showed that chlorophyll a was always more abundant with respect to chlorophyll b, underlining the major involvement of chlorophyll a in the photosynthetic process. The maximum content of chlorophyll a, chlorophyll b, and total carotenoids was observed in stage I or stage II, depending on the cultivar. Conversely, regardless the cultivar, the lowest concentrations of the analyzed pigments were always found in stage III. Among the cultivars, Eletta Campana cultivar was characterized by the highest values of all the analyzed pigments in all the harvesting stages, whereas Tiborszallasi cultivar showed the opposite trend. Considering the chl a/chl b ratio, all samples showed values above 1.3, revealing that hemp plants were grown in light exposition conditions at all the considered stages. Additionally, the chl (a+b)/car ratio indicated the light exposition conditions of the plants; moreover, the obtained values showed that, until the end of September, plants did not reach their senescence phase, except for Tiborszallasi cultivar (ratio value of 3.20). It was noteworthy that the quantitative trend of chlorophylls and carotenoids during the considered stages was strictly correlated. In particular, an increase in chlorophylls generally coincided with an increment of carotenoids, too, although with different rates. This correlation could be explained since chlorophylls and carotenoids synthesis were characterized by common pathways [29,30].

Regarding the total phenolic content, the lowest TPC value was measured in Fibranova in stage I, whereas the highest one was detected in Tiborszallasi in stage II. The TPC results suggested a different behavior of the investigated cultivars. Fibranova, Tisza, Tiborszallasi, and Eletta Campana presented a similar trend with an increment of TPC values in stage II, followed by a decrease (with different slopes) in stage III. Carmagnola and Kompolti, indeed, had a decrement in the last harvesting stage. Antal cultivar showed a unique trend, with a decrease in TPC values during the three harvesting stages. DPPH and ABTS showed different profiling of the cultivars during the harvesting stages. Carmagnola, Fibranova, Tisza, and Eletta Campana showed a similar trend for DPPH and ABTS assays with the highest values in stage II. Antal, here too, showed a unique trend with the decrease in the %I for both tests. Kompolti presented a different trend for the antioxidant activity tests; in the DPPH assay, its trend was the same as the Antal cultivars, whereas, in the ABTS assay, the trend was that of the other cultivars. In a previous work [31], the TPC and the antioxidant activity (DPPH assay) of Carmagnola, Antal, Kompolti, and Tiborszallasi cultivars were determined. However, since the sample treatment, the extraction procedure, and the expression of the results were different from those of the present work, and it was not possible to make a comparison. Among the analyzed classes of compounds, polyphenols were, of course, involved in the antioxidant activity of hydroalcoholic hemp extracts since this activity was well recognized for these compounds [32,33]. In particular, hemp inflorescences showed to be characterized by phenolic acids and flavonoids as the main polyphenolic compounds [31,34]. Moreover, also cannabinoids, mainly THC and CBD, proved to be characterized by antioxidant activity [35,36]. Since the chemical profile of hemp inflorescences was very complex, it could be not easy to correlate the antioxidant activity trend of hemp extracts with that of specific chemical compounds, which was also shown by literature data [37]. In fact, the Pearson correlation coefficient (Table S1) not always showed a strong correlation between TPC and antioxidant activity assays. However, it could be supposed that polyphenols and cannabinoids were strongly involved in the antioxidant activity of hemp inflorescences, which was supported by the obtained results. In particular, for the analyzed cultivars, the highest antioxidant activity was generally observed in stage II, as well as the content of cannabinoids and total polyphenols. Moreover, Antal cultivar, whose antioxidant activity was higher in stage I, was also characterized by the highest contents of polyphenols and cannabinoids, particularly CBD, in this period.

As for the metabolite profile of hemp inflorescences, it was possible to observe analogies and differences among cultivars and harvesting stages. Among amino acids, asparagine was found to be the most abundant in all cultivars, mainly in the last two harvesting stages, with the highest content measured in Kompolti cultivar in stage II (31%). Sucrose content was very high in all cultivars in stage I, with the highest concentration found in Tisza cultivar (38%), whereas in stage III its content was very low, reaching the lowest value in Tiborszallasi cultivar (0.6%). Malic acid was the main organic acid, with the highest concentration measured in Kompolti cultivar in stage III. These metabolites can affect the sensorial properties of hemp infusions and beverages that can be used for food or pharmacological purposes [38]. The PCA analysis of NMR data showed that in general, regardless of the cultivar, metabolite profiles could be differentiated owing to the harvesting stage. In particular, samples harvested in stage I were characterized by high contents of sucrose and myo-inositol, whereas the ones harvested in stage II showed higher levels of succinic acid, alanine, and essential amino acids. Samples harvested in stage III were distinguished by high levels of monosaccharides, choline, trigonelline, malic acid, formic acid, and some amino acids. In a previous work [12], the NMR metabolite profile of four monoecious hemp cultivars was determined by using the same analytical protocol of the present work. Comparing the quantitative results obtained for both monoecious and dioecious cultivars in September (considering the 14 September data for the present work), it was possible to observe that the two groups presented peculiar metabolites (Figure S1). In particular, higher levels of fructose, formic acid, malic acid, choline, and trigonelline were observed in monoecious cultivars, whereas dioecious cultivars were characterized by higher amounts of glucose and some amino acids: valine, threonine, alanine, and asparagine. The obtained results allowed us to observe how, basing on the cultivar and/or the harvesting stage, hemp inflorescences were characterized by a peculiar chemical profile suitable for several industrial applications. For instance, mid-September harvesting (stage II) was the most effective to obtain high concentrations of CBD and polyphenols; suggesting this harvesting stage as the main effective to obtain inflorescences with high pharmaceutical and nutraceutical properties. However, for Antal cultivar, the proper period to obtain inflorescences rich in bioactive compounds was early September (stage I). Moreover, secondary metabolites, such as sugars and organic acids, could affect the sensorial properties of hemp-based products, such as infuses. In this context, the Tisza cultivar was characterized by high levels of sugars in stage I and stage III harvesting periods, whereas Kompolti was particularly rich in malic acid in stage III.

## 4. Materials and Methods

### 4.1. Reagents and Chemicals

Gradient grade water, acetonitrile, chloroform, methanol, and formic acid were purchased from Merck Life Science (Milano, Italy). Cannabinoid reference standards dissolved in methanol, namely, cannabidivarin (CBDV, 1 mg/mL), cannabigerol (CBG, 1 mg/mL), cannabidiol (CBD, 1 mg/mL), cannabidiolic acid (CBDA, 1 mg/mL), cannabinol (CBN, 1 mg/mL), (–)-Δ9-tetrahydrocannabinol ((–)-Δ9-THC, 1 mg/mL), Δ9-tetrahydrocannabinolic acid ((–)-Δ9-THCA, 1 mg/mL), (–)-Δ8- tetrahydrocannabinol ((–)-Δ8-THC, 1 mg/mL), and cannabichromene (CBC, 1 mg/mL) with purity ≥ 99%, were purchased from Merck Life Science (Milano, Italy). Deuterated water (D_2_O) 99.97 atom% of deuterium and 3-(trimethylsilyl)-propionic-2,2,3,3-d_4_ acid sodium salt (TSP) were purchased from Euriso-Top (Saclay, France). Magnesium oxide (MgO), potassium phosphate monobasic (KH_2_PO_4_), and potassium phosphate dibasic (K_2_HPO_4_) 2,2-azino-bis (3-ethyl-benzothiazoline-6-sulfonic acid) diammonium salt (ABTS), 2,2-diphenyl-1-picrylhydrazyl (DPPH), Folin-Ciocalteu reagent, gallic acid (GA), and sodium carbonate (Na_2_CO_3_) were purchased from Merck Life Science (Milano, Italy).

### 4.2. Hemp Plant Material

Fresh inflorescences of seven dioecious female *Cannabis sativa* L. cultivars (Carmagnola, Fibranova, Eletta Campana, Antal, Tiborszallasi, Kompolti, and Tisza) were provided by the Canapa Live cultural association. They were grown in experimental fields located in the North Lazio area (Italy, Rome) during the 2018 cultivation year. Carmagnola, Fibranova, and Eletta Campana are Italian cultivars, Antal is a cultivar from the Czech Republic, whereas Tisza, Tiborszallasi, and Kompolti are cultivars from Hungary. The same agronomical practices, previously described in [12], were used for plant cultivation. The inflorescences sampling was carried out by applying the following systematic pattern: for each cultivar, 25 plants were collected in the central part of the cultivation area by cutting the upper part (30 cm) of the stem. Considering the late (Antal, Tisza, Tiborszallasi, Kompolti) and very late (Carmagnola, Fibranova, Eletta Campana) vegetative cycles of the selected cultivars, female inflorescences were harvested in the following three stages: 4 September (stage I), 14 September (stage II), and 30 September (stage III) reaching from beginning of flowering to the end of flowering/beginning of seed formation. After harvesting, fresh inflorescences were ground under N_2_ and stored at −80 °C until analysis. Analyzes were carried out within ten days of storage. Note that, since 2019, Carmagnola, Fibranova, and Antal cultivars were banned from the EU Plant Variety Database [5] because of their unstable THC content.

### 4.3. Cannabinoids Content by UHPLC Analyses

For each sample, 4 g of ground fresh inflorescences were dried at 70 °C for 48 h before extraction. After drying, 100 mg of inflorescences were extracted with 4 mL of methanol [39,40] in an ultrasonic bath for 30 min. The residual pellet was re-extracted again two times. Each extract was then filtered under vacuum conditions, diluted to 5 mL with methanol, filtered with a 0.45 μm PTFE membrane, and finally analyzed.

Analyses were carried out by using an Ultimate 3000 ultra-high-performance liquid chromatography (UHPLC; Thermo Fisher Scientific) (Rodano, Italy), with a binary gradient system, an automatic injector, a thermostatic column compartment, and a diode array detector. The system was controlled by Chromeleon Chromatography Data System software (Thermo Fisher Scientific, 1.0.5. v, Waltham, MA, USA, 2018. All separations were performed by using a Titan C 18 column (10 cm × 3 cm, 1.9 μm, Sigma Aldrich (Milan, Italy) with a mobile phase composed of 0.1% formic acid in both (A) water and (B) acetonitrile. The total run time was 22 min, and the chromatographic conditions were set as follows: 0–14 min from 65 to 100% B, 15–16 min isocratic elution with 100% B, 17–22 min from 100 to 65% B. The flow rate was 0.5 mL/min. The column temperature was set at 35 °C. A volume of 10 μL was injected. The PDA detector was set to 214 nm wavelength.

To prepare the calibration curve, standard cannabinoid solutions of CBDV, CBD, CBC, CBDA, CBG, CBN, Δ8-THC, Δ9-THC, and Δ9-THCA were prepared in the concentration range from 0.001 to 0.05 mg/mL.

Regression lines were calculated using the least squares method, and linearity was expressed by the determination coefficient (R^2^). For each calibration curve, the R^2^ value was always greater than 0.998, underlining good linearity.

Cannabinoid concentrations were expressed as percentage (*w/w*) ± SD (standard deviation; Table 2). Three replications were made for each sample.

### 4.4. Untargeted NMR Analysis and Multivariate Statistical Analysis

For the untargeted analysis, the fresh plant material was extracted by using the Bligh–Dyer protocol [41] already described in detail [12]. The dried hydroalcoholic extracts were solubilized in 0.75 mL of 400 mM phosphate buffer/D_2_O, containing a 1 mM TSP as internal standard, and then transferred into a 5 mm NMR tube. NMR analyses were carried out on a Bruker AVANCE 600 spectrometer operating at the proton frequency of 600.13 MHz and equipped with a Bruker multinuclear z-gradient 5 mm probe head (Milan, Italy). ^1^H spectra were registered using the following parameters: 28 °C sample temperature, 256 transients, suppression of residual water signal (HDO) using a pre-saturation, 5 s recycle delay, 45° pulse of 7.5 µs, and 32 K data points. Two-dimensional (2D) experiments (^1^H-^1^H TOCSY, ^1^H-^13^C HSQC, and ^1^H-^13^C HMBC) were carried out using the same experimental conditions previously reported [42]. Spectra were processed using TopSpin software (version 4.0.6, Billerica, MA, USA, 2017). For quantitative analysis, the integrals of the corresponding selected resonances in ^1^H NMR spectra (Table 2) were measured with respect to the internal standard TSP, allowing the molar concentration and the corresponding weight to be calculated. The amount of each metabolite was expressed as weight percentage with respect to the total weight of all quantified metabolites. Each sample was extracted and analyzed three times. The 21 quantified metabolites (Figure 2 and Figure 3) in the hydroalcoholic phase were used as variables for the principal component analysis (PCA) by using MATLAB software (version R2020b). Before the analysis, the selected variables were processed using autoscaling and then mean centered.

### 4.5. Spectrophotometric Analysis of Chlorophylls and Total Carotenoids

Pigments extraction was carried out according to Mizliack–Solovchenko, with some modifications [43]: 30 mg of the sample was homogenized with mortar and pestle in 6 mL of a chloroform–methanol (2:1, *v/v*) mixture, together with 20 mg of MgO, in order to neutralize plant acids and prevent pheophytin formation [22]. After filtration, a volume of distilled water equal to the 20% of the organic extract volume was added, and the emulsion was centrifuged for 20 min (20 °C, 2500× *g*). The organic phase, which contained chlorophylls and carotenoids, was separated from the hydroalcoholic one and then analyzed. The absorption spectrum was acquired by an ONDA spectrophotometer UV-30 SCAN (spectral resolution 0.1 nm) in the wavelength range of 350–800 nm at 25 °C (sipper system A-100 series) (Milan, Italy). The absorbance values measured at 480, 648, and 666 were considered. The concentrations (µg/mL) of chlorophyll a (Chl a), chlorophyll b (Chl b), and total carotenoids (Car) were calculated according to Wellburn equations [44], where A is the measured absorbance at the selected wavelength:

C_Chl a_ = 11.47 × A666 − 2 × A648 (1)

C_Chl b_ = 21.85 × A648 − 4.53 × A666 (2)

C_car_ = (1000 × A480 − 1.33 × C_Chl a_ − 23.93 × C_Chl b_)/202 (3)

The obtained concentrations were then converted in mg/g ± SD (standard deviation) and reported as the means of three replications. Chl a/chl b and chl (a+b)/car ratios were also determined. Significant differences in the levels of the analyzed compounds among the cultivars at the same stage and among the same cultivar in different stages were evaluated by a one-way analysis of variance (one-way ANOVA), followed by Bonferroni’s Multiple Comparison Post Test.

### 4.6. Spectrophotometric Analysis of Total Phenolics Content and Antioxidant Activity

The extraction of phenolic compounds was carried out following a protocol previously described [45], with some modifications. In particular, 0.5 g of fresh hemp inflorescences were added to 2.5 mL of MeOH/H_2_O 7:3 *v/v* solution. Samples were then shacked for 1 min, sonicated in an ultrasonic bath for 5 min, and finally centrifuged for 10 min at 2500× *g*. After centrifugation, the supernatant was separated from the residual pellet that was extracted again using the same conditions described above. Supernatants obtained in both extractions were united, obtaining a final volume of 5 mL.

Total phenolics content (TPC) was determined using the Folin–Ciocalteu method [45], optimizing the protocol for hemp inflorescences: 1 mL of hydroalcoholic extract was added to 0.25 mL of Folin–Ciocalteu reagent and 0.5 mL of Na_2_CO_3_ (7.5% *w/v*) in a 10 mL volumetric flask, reaching the final volume with water. Each sample was stored in the dark for 45 min at room temperature, and the spectrophotometric analysis (Lenway 6705 UV-Vis spectrophotometer) (Milan, Italy) was performed at 750 nm. TPC was expressed as milligrams of gallic acid equivalents (GAE)/kg: a calibration curve was created, with a concentration range from 15 to 500 mg/L (R^2^ = 0.9925). The measurements were carried out in triplicate for each sample and reported as mean value ± SD.

The antioxidant activity of hemp hydroalcoholic extracts was determined through DPPH and ABTS assays. For the DPPH assay, 1.5 mL of reagent were added to 1 mL of hydroalcoholic extract and left in the dark at room temperature. After 30 min, the absorbance of the solution was recorded. For the ABTS assay, 3.6 mL of reagent were added to 0.4 mL of hydroalcoholic extract and left in the dark at room temperature. After 15 min the absorbance of the solution was recorded. The reduction of radical concentration was evaluated by measuring the absorbance at 515 nm (for DPPH assay) and 734 nm (for ABTS assay), as previously reported [45]. Results were expressed as inhibition rate (I%) and were calculated according to the following equation:

I% = (A_0_ − A_f_)/A_0_ × 100

where A_0_ is the initial absorbance of the radical cation and A_f_ is the absorbance of the radical cation after the extract addition. The assays were carried out in triplicate for each sample and reported as mean value ± SD. Significant differences in the levels of the analyzed compounds among the cultivars at the same stage and among the same cultivar in different stages were evaluated by one-way analysis of variance (one-way ANOVA), followed by Bonferroni’s Multiple Comparison Post Test. Pearson correlation coefficients were also evaluated and reported in Table S1.

## 5. Conclusions

The present study showed that the *Cannabis sativa* L. inflorescences of each analyzed hemp cultivar presented a peculiar chemical profile that could be affected by the harvesting stage. The knowledge of the inflorescences chemical profile was a prerequisite to promote their use in several industrial fields, being a significant source of compounds with specific sensorial/nutraceutical/pharmaceutical properties.

## Figures and Tables

**Table 1:** Cannabinoid concentrations by ultra-high-performance liquid chromatography (UHPLC analysis (% (*w/w*) ± SD) reported for each cultivar at three harvesting stages (stage I = 4 September, stage II = 14 September, stage III = 30 September). Analyses were carried out in triplicate.

Cultivar	Harvesting Stage	(–)-Δ9-THC	CBD	(–)-Δ9-THCA	CBDA	CBG	CBDV	CBC	CBN
Carmagnola	stage I	0.24 ± 0.02	3.10 ± 0.40	-	2.02 ± 0.30	0.13 ± 0.01	-	0.19 ± 0.02	-
	stage II	0.23 ± 0.01	3.12 ± 0.21	-	1.52 ± 0.02	0.17 ± 0.01	^-^	0.18 ± 0.01	-
	stage III	0.16 ± 0.01	3.82 ± 0.82	-	-	0.16 ± 0.01	-	0.13 ± 0.01	-
Fibranova	stage I	0.19 ± 0.01	2.05 ± 0.03	-	0.88 ± 0.04	0.18 ± 0.01	-	0.21 ± 0.01	-
	stage II	0.26 ± 0.01	2.97 ± 0.13	-	1.57 ± 0.07	0.18 ± 0.01	0.41 ± 0.01	0.33 ± 0.01	-
	stage III	0.11 ± 0.01	2.31 ± 0.30	-	-	-	-	-	0.11 ± 0.03
Kompolti	stage I	0.28 ± 0.01	2.90 ± 0.20	-	1.40 ± 0.10	0.15 ± 0.01	-	0.18 ± 0.01	-
	stage II	0.30 ± 0.01	5.09 ± 0.06	-	1.55 ± 0.02	0.18 ± 0.02	-	0.31 ± 0.01	-
	stage III	0.14 ± 0.01	4.64 ± 0.65	-	-	0.23 ± 0.01	-	0.15 ± 0.04	-
Tisza	stage I	0.193 ± 0.005	2.400 ± 0.100	-	1.280 ± 0.040	0.210 ± 0.010	-	0.17 ± 0.01	-
	stage II	0.250 ± 0.010	4.160 ± 0.220	-	1.910 ± 0.010	0.160 ± 0.010	-	0.21 ± 0.01	-
	stage III	0.061 ± 0.001	4.600 ± 0.090	-	-	0.091 ± 0.002	-	-	-
Antal	stage I	0.25 ± 0.01	4.30 ± 0.40	-	1.60 ± 0.30	0.22 ± 0.01	-	-	-
	stage II	0.14 ± 0.01	1.55 ± 0.15	-	1.11 ± 0.10	0.12 ± 0.01	-	-	-
	stage III	0.12 ± 0.01	1.96 ± 0.15	-	-	0.10 ± 0.01	0.09 ± 0.01	0.03 ± 0.01	-
Tiborszallasi	stage I	0.21 ± 0.01	2.76 ± 0.04	-	1.24 ± 0.03	0.24 ± 0.01	-	0.18 ± 0.01	-
	stage II	0.51 ± 0.07	4.54 ± 0.22	0.09 ± 0.01	1.87 ± 0.06	0.21 ± 0.01	-	0.22 ± 0.02	-
	stage III	0.13 ± 0.01	3.79 ± 0.41	-	-	0.12 ± 0.02	-	0.15 ± 0.02	-
Eletta Campana	stage I	0.17 ± 0.01	1.50 ± 0.10	-	1.00 ± 0.10	0.13 ± 0.01	-	-	-
	stage II	0.190 ± 0.01	2.04 ± 0.01	-	1.12 ± 0.02	0.12 ± 0.01	-	0.11 ± 0.01	-
	stage III	0.06 ± 0.01	1.37 ± 0.12	-	-	0.04 ± 0.01	-	0.05 ± 0.01	0.04 ± 0.01

**Table 2:** Chlorophyll a, chlorophyll b, and total carotenoid concentrations by spectrophotometric analysis (mg/g of fresh inflorescences ± SD) reported for each cultivar at three harvesting stages (stage I = 4 September, stage II = 14 September, stage III = 30 September). Chl a/b and Chl (a+b)/car ratios are also reported. Analyses were carried out in triplicate.

Cultivar	Harvesting Stage	Chlorophyll a	Chlorophyll b	Chl. a/b	Total Caroteoids	Chl. (a+b)/Car
Carmagnola	stage I	0.527 ± 0.050 ^§,e,c^	0.337 ± 0.071 ^§,b,d,f^	1.83 ^§,f^	0.210 ± 0.023	4.03
	stage II	0.519 ± 0.004	0.272 ± 0.002	1.93	0.176 ± 0.002 ^§,f^	4.49 ^§,c,d,f,g^
	stage III	0.425 ± 0.008 *	0.235 ± 0.002	1.81	0.158 ± 0.002	4.16 ^§,c,d,e,f,g^
Fibranova	stage I	0.540 ± 0.011 ^§,c,e^	0.275 ± 0.004	2.27 ^§,f,g^	0.201 ± 0.004	3.79
	stage II	0.683 ± 0.011 ^§,a,c,d,e,f,g^	0.362 ± 0.004 ^§,a,c,d,e,f^	1.89	0.223 ± 0.003 ^§,a,c,e,f^	4.68 *^,^^§,a,c,d,f,g^
	stage III	0.271 ± 0.006	0.154 ± 0.001	1.76	0.106 ± 0.002	4.03 ^#,§,f^
Kompolti	stage I	0.618 ± 0.002 ^§,a,b,d,e,f^	0.330 ± 0.009 ^§,a,b,d,e,f^	1.87	0.220 ± 0.001	4.32
	stage II	0.525 ± 0.004 ^§,f^	0.290 ± 0.001	1.79	0.196 ± 0.002 ^§,a,f^	4.15
	stage III	0.394 ± 0.002	0.219 ± 0.007	1.80	0.161 ± 0.001	3.81 *^,^^#,§,f^
Tisza	stage I	0.484 ± 0.003	0.241 ± 0.005	2.01 ^§,c,f,g^	0.176 ± 0.001	4.13
	stage II	0.631 ± 0.006 *^,^^§,a,c,e,f^	0.324 ± 0.004 *^,^^§,a,e,f^	1.97 *	0.223 ± 0.003 *^,^^§,a,c,e,f^	4.29
	stage III	0.261 ± 0.004 *^,^^#^	0.161 ± 0.001 *,^#^	1.62 *	0.111 ± 0.002 *^,^^#^	3.79 *^,^^#,§,f^
Antal	stage I	0.565 ± 0.008 ^§,c,e^	0.303 ± 0.008 ^§,d,f^	1.86 ^§,f,g^	0.218 ± 0.001 ^§,e,c^	3.98
	stage II	0.580 ± 0.001 ^§,a,c,f^	0.304 ± 0.003 ^§,a,f^	1.93	0.188 ± 0.001	4.70 ^§,a,c,d,f,g^
	stage III	0.354 ± 0.002 *^,^^#^	0.192 ± 0.003 *^,^^#^	1.84	0.142 ± 0.001	3.85 ^§,f^
Tiborszallasi	stage I	0.335 ± 0.002	0.175 ± 0.003	1.91	0.129 ± 0.001	3.97
	stage II	0.449 ± 0.010 *	0.256 ± 0.002 *	1.73	0.162 ± 0.003 *	4.34 *^,^^§,c^
	stage III	0.218 ± 0.001 *^,^^#^	0.122 ± 0.001 *^,^^#^	1.79	0.106 ± 0.000 *^,^^#^	3.20 *^,^^#^
Eletta Campana	stage I	0.861 ± 0.010 ^§,a,b,c,d,e,f^	0.432 ± 0.004 ^§,a,b,c,d,e,f^	1.99	0.317 ± 0.003 ^§,d,e,f^	4.08
	stage II	0.637 ± 0.001 *^,^^§,a,c,e,f^	0.366 ± 0.002 *^,^^§,a,c,d,e,f^	1.73	0.232 ± 0.001 *^,^^§,a,c,e,f^	4.32 *
	stage III	0.516 ± 0.004 *^,^^#,§,d,f^	0.275 ± 0.004*^,^^#,§,d,f^	1.88	0.205 ± 0.001 *^,^^#,§,f^	3.86 *

* *p* < 0.001, significantly different from the level in the same cultivar in stage I; ^#^ *p* < 0.001, significantly different from the level in the same cultivar in stage II; ^§^ *p* < 0.001, significantly different than the other cultivars in the same stage; ^a^ vs. Carmagnola; ^b^ vs. Fibranova; ^c^ vs. Kompolti; ^d^ vs. Tisza; ^e^ vs. Antal; ^f^ vs. Tiborszallasi; ^g^ vs. Eletta Campana (one-way ANOVA, followed by Bonferroni’s Multiple Comparison Post Test).

**Table 3:** Total phenolic content (TPC) and antioxidant activity by spectrophotometric analysis (mg/g of fresh inflorescences or inhibition rate (I%) ± SD) reported for each cultivar in different harvesting stages (stage I = 4 September, stage II = 14 September, stage III = 30 September). Analyses were carried out in triplicate.

Cultivar	Harvesting Time	TPC (mg GAE/Kg FW)	DPPH (I%)	ABTS (I%)
Carmagnola	stage I	1.95 ± 0.06	72.87 ± 0.17	88.03 ± 0.39
	stage II	2.00 ± 0.06	78.74 ± 0.07	90.63 ± 0.74 ^§,c,f^
	stage III	1.88 ± 0.08	75.38 ± 0.37	86.54 ± 0.89
Fibranova	stage I	1.65 ± 0.06	72.27 ± 0.17	85.56 ± 0.75 ^§,e^
	stage II	2.38 ± 0.12	78.68 ± 0.15	92.68 ± 0.82 ^§,c,f^
	stage III	2.01 ± 0.10	71.67 ± 0.43	86.66 ± 0.21
Kompolti	stage I	2.55 ± 0.08	75.42 ± 3.52	86.91 ± 0.39
	stage II	2.57 ± 0.10 ^§,e^	75.57 ± 0.62	87.89 ± 0.69 *
	stage III	2.11 ± 0.06	72.63 ± 0.76	81.88 ± 0.33 ^#^
Tisza	stage I	2.16 ± 0.09	73.17 ± 1.16	85.98 ± 0.29 ^§,e,c^
	stage II	2.30 ± 0.10	75.29 ± 0.15	95.24 ± 0.16 *^,§,c,d^
	stage III	2.02 ± 0.06	71.97 ± 0.57	84.36 ± 0.42 *
Antal	stage I	1.84 ± 0.04	76.03 ± 1.34	95.35 ± 0.42
	stage II	1.67 ± 0.07	75.08 ± 0.81	84.84 ± 0.90 ^§,c,d,f^
	stage III	1.54 ± 0.08	74.56 ± 0.52	84.56 ± 0.17 ^§,a,b,c,d,f^
Tiborszallasi	stage I	2.41 ± 0.08	75.39 ± 0.86	87.32 ± 0.31
	stage II	2.68 ± 0.13 ^§,e^	80.68 ± 1.07 ^§,d^	88.03 ± 1.05 *
	stage III	2.42 ± 0.09 ^§,a,e^	79.86 ± 0.79	84.18 ± 0.74 ^#^
Eletta Campana	stage I	1.80 ± 0.0985.63	74.40 ± 0.51	91.67 ± 1.19 ^§,e^
	stage II	2.10 ± 0.07	77.32 ± 0.44	93.05 ± 1.12 ^§,c,f^
	stage III	2.05 ± 0.10	70.44 ± 3.66	92.40 ± 0.46 ^§,a,b,c,d,f^

* *p* < 0.001, significantly different from the level in the same cultivar in stage I; ^#^ *p* < 0.001, significantly different from the level in the same cultivar in stage II; ^§^ *p* < 0.001, significantly different than the other cultivars in the same stage; ^a^ vs. Carmagnola; ^b^ vs. Fibranova; ^c^ vs. Kompolti; ^d^ vs. Tisza; ^e^ vs. Antal; ^f^ vs. Tiborszallasi (one-way ANOVA, followed by Bonferroni’s Multiple Comparison Post Test).

**Table 4:** Compounds, with relative ^1^H NMR signals (ppm), quantified in *Cannabis sativa* L. inflorescences Bligh–Dyer hydroalcoholic extracts. Analyses were carried out in triplicate.

Compound	ppm	Compound	ppm
Isoleucine	1.02	Choline	3.21
Valine	1.05	Myo-inositol	3.30
Threonine	1.34	Fructose	4.04
Alanine	1.49	Malic acid	4.30
Proline	2.00	β-Glucose	4.66
Glutamic acid	2.07	α-Glucose	5.25
GABA	2.30	Sucrose	5.42
Succinic acid	2.41	Phenylalanine	7.43
Glutamine	2.46	Tryptophan	7.53
Aspartic acid	2.83	Formic acid	8.47
Asparagine	2.89	Trigonelline	9.12

## Data Availability

Not applicable.

## Supplementary Materials

The following are available online. Figure S1. PCA maps obtained comparing September inflorescences samples of monoecious and dioecious Cannabis sativa L. cultivars: (A) scores plot and (B) loadings plot relative to PC1 and PC2 (PCs); PC1 and PC2 represent 49.0% and 25.5% of the total variance, respectively. Table S1. Pearson correlation coefficient (r) between total polyphenolic compounds and antioxidant activity. * Significant at *p* ≤ 0.05.

## Sample Availability

Samples are available from the authors.
