# Supplementary material for: A Multimethodological Characterization of Cannabis sativa L. Inflorescences from Seven Dioecious Cultivars Grown in Italy: The Effect of Different Harvesting Stages
Source: Molecules. 2021 May 14;26(10):2912. doi: 10.3390/molecules26102912 (PMC8156653; doi:10.3390/molecules26102912)
Supplement: Supplementary file 1 [file molecules-26-02912-s001.zip › molecules-1204964-supplementary.pdf]

A Multimethodological Characterization of *Cannabis sativa* L. Inflorescences from Seven Dioecious Cultivars Grown in Italy: The Effect of Different Harvesting Stages

Mattia Spano [1,†], Giacomo Di Matteo [1,†], Cinzia Ingallina [1], Bruno Botta [1], Deborah Quaglio [1], Francesca Ghirga [1], Silvia Balducci [1], Silvia Cammarone [1], Enio Campiglia [2], Anna Maria Giusti [3], Giuliana Vinci [4], Mattia Rapa [4], Salvatore Ciano [4], Luisa Mannina [1,*] and Anatoly P. Sobolev [5]

1 Department of Chemistry and Technology of Drugs, Sapienza University of Rome, Piazzale Aldo Moro 5, 00185 Rome, Italy; mattia.spano@uniroma1.it (M.S.); giacomo.dimatteo@uniroma1.it (G.D.M.); cinzia.ingallina@uniroma1.it (C.I.); bruno.botta@uniroma1.it (B.B.); deborah.quaglio@uniroma1.it (D.Q.); francesca.ghirga@uniroma1.it (F.G.); silvia.balducci@uniroma1.it (S.B.); silvia.cammarone@uniroma1.it (S.C.)

2 Department of Agricultural and Forest Sciences, University of Tuscia, Via San Camillo de Lellis snc, 01100 Viterbo, Italy; campigli@unitus.it

3 Department of Experimental Medicine, Sapienza University of Rome, P.le Aldo Moro 5, 00185 Rome, Italy; annamaria.giusti@uniroma1.it

4 Department of Management, Sapienza University of Rome, via del Castro Laurenziano 9, 00161 Rome, Italy; giuliana.vinci@uniroma1.it (G.V.); mattia.rapa@uniroma1.it (M.R.); salvatore.ciano@uniroma1.it (S.C.)

5 Institute for Biological Systems, Magnetic Resonance Laboratory "Segre-Capitani", CNR, Via Salaria Km 29.300, 00015 Monterotondo, Italy; anatoly.sobolev@cnr.it

* Correspondence: luisa.mannina@uniroma1.it;

† These authors contributed equally to this work.

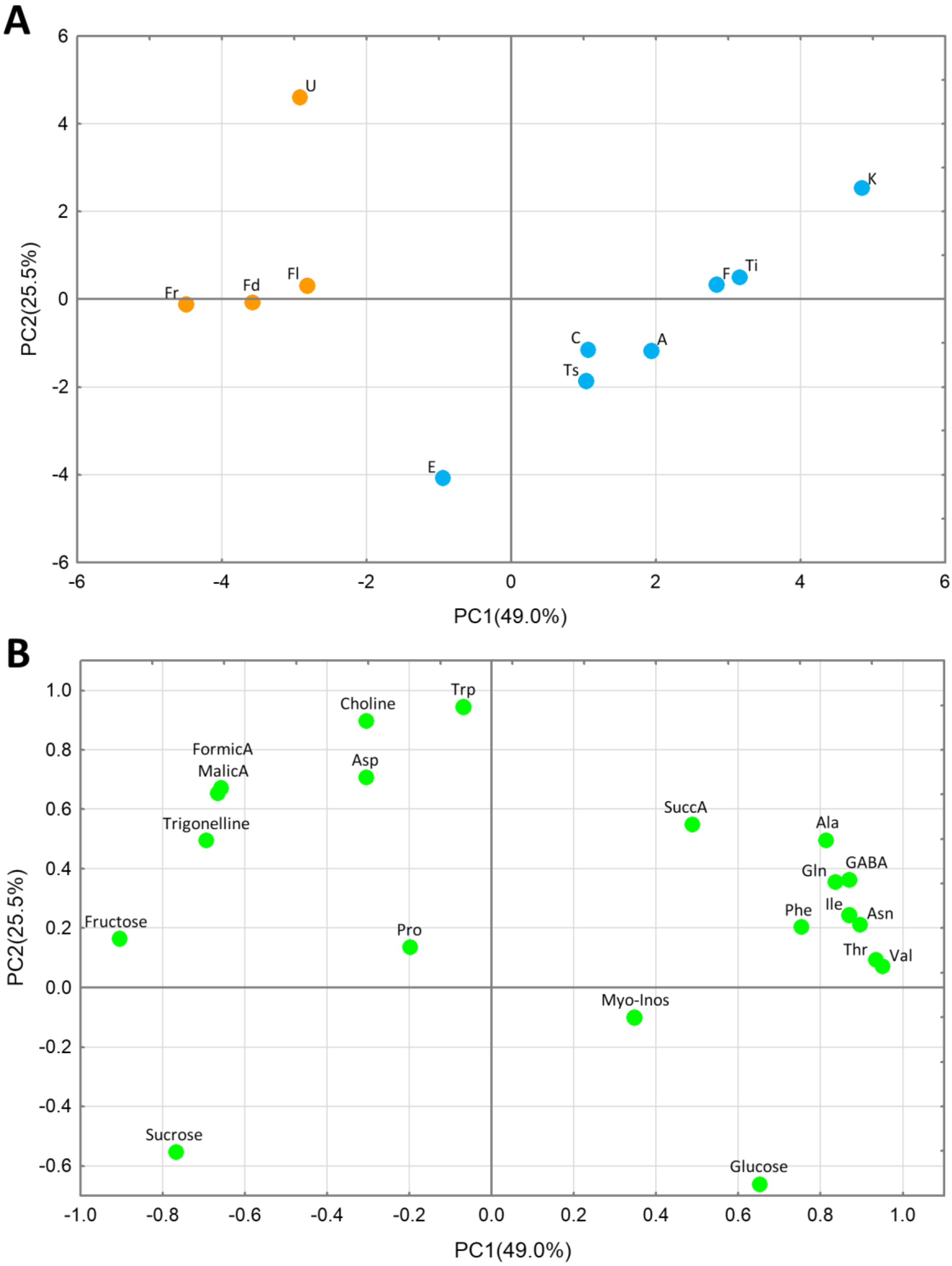

Figure S1. PCA maps obtained comparing September inflorescences samples of monoecious and dioecious *Cannabis sativa* L. cultivars: (A) scores plot and (B) loadings plot relative to PC1 and PC2 (PCs); PC1 and PC2 represent 49.0% and 25.5% of the total variance, respectively.

Table S1. Pearson correlation coefficient (r) between total polyphenolic compounds and antioxidant activity. *Significant at $p \leq 0.05$.

	**stage I**		**stage II**		**stage III**	
	DPPH	ABTS	DPPH	ABTS	DPPH	ABTS
**Kompolti Tisza Tiborszallasi**	-0,1930	-0,7012	0,7743	-0,9113*	0,8196*	0,3324
**Carmagnola Antal**	-0,6214	0,9733*	0,8923	-0,9352	-0,3155	0,7053
**Fibranova Eletta Campana**	0,8605	0,4858	0,8063	0,8478	-0,6590	0,9338
